# Intravitreal Dexamethasone Implant (Ozurdex) for Ocular Toxocariasis

**DOI:** 10.1155/2024/6685092

**Published:** 2024-07-15

**Authors:** Yongwei Zhou, Fangyuan Zhen, Jiahui Wu, Shasha Wang, Xiaoyan Lu, Ge Yang, Zhirou Hu, Fei Chen, Qiuming Li, Shuqian Dong

**Affiliations:** Department of Ophthalmology The First Affiliated Hospital of Zhengzhou University Henan Provincial Ophthalmic Hospital, Zhengzhou, China

## Abstract

This study aimed to evaluate the efficacy and safety of intravitreal dexamethasone implants in the treatment of ocular toxocariasis (OT). A retrospective analysis was performed on 6 cases in which laboratory tests diagnosed OT. All patients were administered with intravitreal dexamethasone implants with or without vitrectomy. The average follow-up time was 19.7 months. All operated eyes achieved anatomic success, and all patients' visual acuity was improved. Five of these six had a visual acuity of 20/100, and three had final acuity of 20/40 or even better. Intravitreal dexamethasone implants can be used to treat different types of OT, which not only effectively control inflammation and improve the patient's vision but also reduce the use of systemic glucocorticoids.

## 1. Introduction

Toxocariasis is a manifestation of a parasitic infection caused by the roundworms *Toxocara canis* or *Toxocara cati* [1, 2]. They are the most prevalent gastrointestinal parasites in dogs and cats; when infected, these animals contaminate the environment through their feces [3]. Ocular toxocariasis (OT) is caused by people accidentally swallowing *Toxocara* eggs, which hatch in the small intestine and release parasitic larvae. These larvae then penetrate the intestinal wall, enter the circulation, and migrate to the eyes, where they cause an inflammatory response [4]. OT is usually unilateral, and its clinical manifestations are diverse, depending primarily on the number of parasites, the site of infection, the host's immune response, and the migration pattern of the larvae [5]. The typical fundus abnormalities are posterior pole granulomas or peripheral granulomas with vitreous traction cords, or moderate to severe vitreous inflammatory reactions with retinal inflammatory masses [6]. The main treatment methods include drug therapy (glucocorticoids and antiparasitic drugs) and surgical treatment [7–9].

Dexamethasone (DEX) is a powerful synthetic member of the glucocorticoid class of steroid drugs that has anti-inflammatory and immunosuppressant activities, 30 times more effective than those of cortisol and 6 times more effective than those of triamcinolone [10]. In recent years, some scholars have proposed to apply the dexamethasone intravitreal implant to OT patients to reduce the use of systemic glucocorticoids [11]. The dexamethasone intravitreal implant (Ozurdex; Allergan, Inc., Irvine, CA) is an approved therapy for diabetic macular edema, retinal vein occlusion, and noninfectious posterior uveitis [12]. The efficacy was demonstrated by improvements in visual acuity and vitreous haze, as well as a decrease in retinal thickness [13]. In this study, we treated patients by injecting DEX implants with or without vitrectomy and studied the therapeutic effect of injection of Ozurdex on patients with ocular toxocariasis. As far as we know, this is the first systematic retrospective study on the treatment of OT with intravitreal DEX implants.

## 2. Materials and Methods

We retrospectively reviewed a case series of 6 patients diagnosed with ocular toxocariasis and treated with intravitreal DEX implants with or without pars plana vitrectomy (PPV) at the First Affiliated Hospital of Zhengzhou University, China, between March 2018 and March 2021. Written informed consent was obtained from the patient or the guardian of the pediatric patient. This study was approved by the Ethics Committee of the First Affiliated Hospital of Zhengzhou University and was conducted in accordance with the Declaration of Helsinki (2021-KY-1241).

Patients underwent routine ophthalmic examination including best-corrected visual acuity (BCVA), intraocular pressure (IOP), a slit-lamp examination with a VOLK 90D lens, scanning laser ophthalmoscopy (Optomap 200Tx; Optos PLC, Dunfermline, UK), and optical coherence tomography (Spectralis OCT; Heidelberg Engineering GmbH, Dossenheim, Germany). Depending on their condition, they were tested with any of the following imaging methods: B-scan ultrasonography (Mylab 8ehd, Esaote, Genoa, Italy) and fundus fluorescein angiography (HRA2; Heidelberg Engineering GmbH, Dossenheim, Germany).

Paired intraocular fluid (IF) from aqueous humor (AH) and serum samples of each participant were also collected. The positive rate of *Toxocara canis-*specific IgG antibody in AH and serum of patients was detected by enzyme-linked immunosorbent assay (ELISA). Serum anti-toxocara IgG greater than 9 are positive, and AH anti-toxocara IgG greater than 3 are positive. All patients were confirmed to be infected by codetection of aqueous humor and serum *Toxocara canis-*specific immunoglobulin G (IgG) antibody and were administered with intravitreal DEX implants for intraocular inflammation.

OT was divided into three types according to clinical manifestations and imaging examinations and references [14, 15]: endophthalmitis type, posterior pole granuloma type, and peripheral granuloma type. For the patients diagnosed as endophthalmitis type, our treatment plan was to inject Ozurdex into vitreous without surgery. For patients diagnosed with posterior and peripheral granuloma type, our treatment plan was vitrectomy combined with Ozurdex. We conducted a retrospective study on the visual acuity and fundus of the patients before and after treatment to analyze treatment methods, results, complications, and visual outcomes. All patients have been followed from 12 to 31 months.

## 3. Results

Six eyes of 6 patients were included in the study. The mean age of the study group was 15.7 years (range: 5–29 years), with gender distribution of two men and four women. All cases were affected unilaterally with three right eyes and three left eyes. A total of four children (<18 years) were featured in this study (range: 5–15 years). Diminution of vision was the most common presenting ocular symptom in both adults and children. All of our patients had an initial visual acuity of 20/100 or worse. Poor visual acuity ≤20/200 was noted in 5 eyes. The mean time from symptom onset to hospital visit was 10.5 months (range: 2 to 36 months). Results for the patients are given in Table 1.

Among the 6 patients, endophthalmitis type, posterior pole granuloma type, and peripheral granuloma type were 2 eyes, 2 eyes, and 2 eyes, respectively. We developed different treatment plans according to the clinical classification of the patients. Of the six patients, one patient was treated only with DEX implant, one patient underwent surgery after DEX implant, and four individuals underwent vitrectomy with DEX implant. For the patients diagnosed as endophthalmitis type, our treatment plan was to inject Ozurdex into vitreous without surgery. Among them, one patient was reinjected with an intravitreal DEX implant 7 months after injection. One patient suffered from inflammation recurrence nine months after the first injection and was found to have retinal detachment with vitreous traction and macular epiretinal membranes. Subsequently, she underwent vitrectomy with membrane peeling. For patients diagnosed with posterior and peripheral granuloma type, our treatment plan was vitrectomy combined with intravitreal DEX implants, with additional scleral buckling in eyes with peripheral granuloma type. Of them, 2 patients received secondary surgery and intravitreal DEX implants. For one patient diagnosed with combined type, only an intravitreal DEX implant was administered because of the patient's heavy vitreous opacity and poor vision.

**Figure 1:**
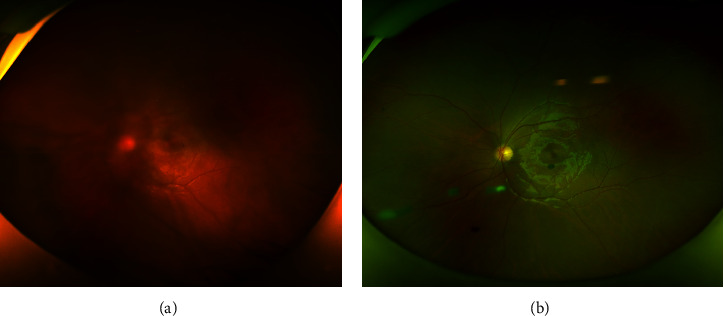
A 12-year-old male diagnosed with the endophthalmitis type. (a) The ultra-widefield fundus photograph showed opacity vitreous in the left eye and it was difficult to see the fundus of the eye. (b) The opacity vitreous improved after the injection, and the fundus was clearly visible. Patient's vision recovered from 20/200 to 20/20.

**Figure 2:**
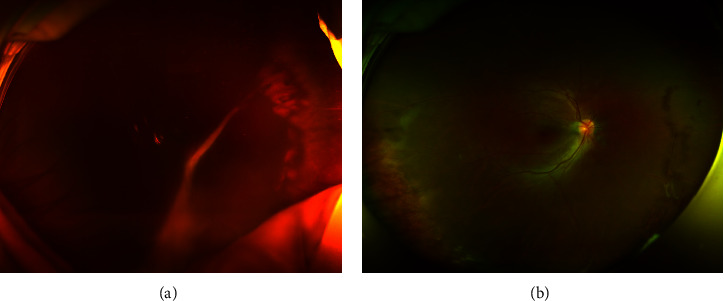
An 11-year-old female with a peripheral granuloma. (a) The ultra-widefield fundus photograph showed diffuse vitreous opacities with peripheral granuloma, a peripheral tractional band attached to the optic disc, and epiretinal membranes in the right eye. (b) After treatment, the anatomy of the retina of the patient's fundus was restored and the inflammation in the eye was reduced. Patient's vision recovered from count finger to 20/40.

The 6 eyes that completed the treatment according to the guidance of ophthalmologists and recorded follow-up were considered for the final visual results. The average follow-up time was 19.7 months (from 12 to 31 months). By the end of the follow-up period, all the operated eyes achieved anatomic success (Figures 1 and 2), and all patients' visual acuity was improved. Five of these six had a visual acuity of 20/100, and three had final acuities of 20/40 or even better (Table 1). During the follow-up period, we did not find any side effects after injection of Ozurdex. The mean IOP was 14.1 mmHg (range 9.0–18.5) at baseline, and there is no increase in intraocular pressure (IOP >21 mmHg or an elevation of IOP of ≥10 mmHg from baseline) in the drug-injected patients. And none of the patients had cataract formation after treatment.

## 4. Discussion

Ocular toxocariasis is a rare worldwide parasitic infectious disease that occurs in both rural and urban areas [16]. It is the main cause of vision loss in children [3]. The most common causes of vision loss are traction retinal detachment, vitreous opacity, cystoid macular edema, and complicated cataract [6, 17]. Its incidence and case characteristics may be different in different areas. Relevant research reports have shown that in most countries such as the United States and in countries in Asia and Africa, the majority of OT patients are children and are related to the history of contact with pets (dog or cat); in South Korea and Japan, the majority of OT patients are adults and are mostly associated with a history of raw food intake [18–20].

The most common symptoms in patients with OT are unilateral vision loss, strabismus, or leukocoria [3]. In our study, five patients came to the clinic with vision loss, including 4 pediatric patients. Three cases presented with retinal detachment, out of which two were children (5 and 15 years of age). This result is consistent with the report of related literature [18]. Compared to adult patients with OT, the symptoms and signs of were more severe in children. The reasons for this may be related to the long onset time of the disease, lack of early independent complaints and untimely discovery by the guardian, the combination of severe traction retinal detachment affecting the macular area, or severe vitreous opacity and lens opacity.

The main goals of the treatment of ocular toxocariasis are to control inflammation, reduce granuloma formation, and improve the patient's vision. It can be treated with medication and surgery. Medical treatment includes glucocorticoids and anthelmintic drugs. However, the use of anthelmintics may cause a strong immune response that, if not adequately controlled, can cause significant structural damage to the eye and more serious complications [21]. As a result, our treatment plan is primarily glucocorticoids combined with vitrectomy. Corticosteroids are used to control inflammation of the vitreous and anterior chamber. Systemic corticosteroids for treatment of ocular disease can cause significant toxicity because of the high concentrations of drugs needed to deliver enough drugs through the tight cellular junctions at the ocular surface or through the blood-retinal barrier [22]. And long-term use of systemic corticosteroids can lead to cumulative complications in multiple major organ systems, such as hyperglycemia, hypertension, osteoporosis, higher susceptibility to infections, psychological problems, and growth suppression [13, 23, 24]. Compared with systemic administration, topical application of glucocorticoids, such as intravitreal triamcinolone, can theoretically provide higher drug concentrations to target tissues and avoid serious systemic complications. Therefore, local glucocorticoids can be used to treat OT to minimize systemic side effects of systemic steroids. Intravitreal injections of triamcinolone acetonide (TA) and sustained-release DEX are commonly used treatment plans for local application of glucocorticoids. TA is a suspension of fine particles, the half-life of it in nonvitrectomized patients was 18.6 days and that in a patient who had undergone a vitrectomy was shorter at 3.2 days, and it may be maintained at a level sufficient for clinical effect for only 3 days [25]. In addition, it has been shown that there are significant differences in the crystal size of TA, and these morphological aspects may have a significant impact on the half-life of the drug [26]. Hence, we attempted to treat OT with the intravitreal dexamethasone implant (Ozurdex). Ozurdex, which contains poly (lactic acid-co-glycolic acid) (PLGA), is a completely biodegradable drug delivery device and can slowly and continuously release the active ingredient of the drug [22]. It can not only achieve adequate intraocular concentration but also greatly reduce the adverse reactions of systemic medication [27, 28]. Many studies have demonstrated the efficacy and safety of Ozurdex in clinical trials for a variety of retinal diseases, such as diabetic macular edema, retinal vein occlusion, and noninfectious uveitis [10, 13, 29].

In this study, we have reported on the efficacy of intravitreal DEX implants in different types of patients with OT. For 2 patients diagnosed with endophthalmitis type, we only injected Ozurdex into the vitreous. One of the patients had a second injection after 7 months and his vision returned to 20/20 after the injection. During the 11 months of follow-up, his vision remained stable. Another patient had a recurrence of inflammation and underwent a vitrectomy. The visual acuity returned to 20/40 after treatment. These results suggest that intravitreal DEX implants can be used as an alternative to systemic glucocorticoids for the treatment of OT patients diagnosed with endophthalmitis type. Because OT inflammation is prone to relapse and the duration of drug action is limited [30], a single injection may not completely control the inflammation. Therefore, we need to closely observe the changes in patient's condition, and if inflammation recurs, we should diagnose and treat it in a timely manner. For patients diagnosed with posterior and peripheral granuloma type, we performed vitrectomy combined with intravitreal DEX implants, with additional scleral buckling in eyes with peripheral granuloma type. Injection of Ozurdex at the end of vitreous surgery for OT can be done safely and simply, and the intravitreal DEX implant lasts longer and has a better safety profile compared to triamcinolone acetonide injections [12, 25, 30]. All of the surgical patients were considered successful, with improved visual acuity and an attached retina postoperatively. This indicates that the intravitreal DEX implant can effectively control inflammation and is an important supplementary therapy for OT patients who develop granuloma or other complications. Furthermore, for patients with peripheral granuloma type, scleral buckling can effectively alleviate the traction of granulomas on the retina and in combination with vitrectomy and Ozurdex injections may achieve better therapeutic results.

However, despite its remarkable role in the treatment of retinal diseases, its side effects should not be ignored. High intraocular pressure and cataracts are the main long-term sequelae identified in large randomized clinical trials [27, 31–33]. According to reports [27, 33], the increase of IOP after Ozurdex injection typically occurs within the first 2 weeks, reaches the peak roughly after 2 months, and then begins to decrease gradually, to reach the preinjection values usually within 6 months. In our study, we analyzed eye function and adverse reactions after Ozurdex injection. Overall, most patients, whether they had surgery or not, had significantly improved visual function after the injections. During the follow-up from 12 to 31 months, we did not find that the patients had elevated intraocular pressure and complicated cataract, even after repeated injections. Although one patient underwent cataract surgery after 7 months of medication, this patient was diagnosed with cataract at the first visit, and his cataract was caused by long-term inflammation.

Our research has several limitations. First, the follow-up time is short. Long-term follow-up is needed to determine the final outcome of eye treatment. These patients should be followed up for a long period of time to observe whether they have recurrent inflammation and symptoms related to recurrent retinal detachment. Second, the sample size is small, so our research may not be representative of all patients with OT in the real clinical environment; we should expand the sample size and continue the research. Third, this study is a retrospective study, and the treatment and follow-up procedures are inconsistent. These limitations should be solved in future research.

## 5. Conclusions

In conclusion, our study provides a novel treatment option that intravitreal DEX implants can be used to treat different types of ocular toxocariasis, which not only effectively control inflammation and improve the patient's vision but also reduce the use of systemic glucocorticoids. Additionally, the slow and sustained release of the drug makes it safer. To the best of our knowledge, our study is the largest case series of OT treated with intravitreal DEX implants.

## Figures and Tables

**Table 1:** Patient characteristics and outcomes.

	Age	Sex	Symptom duration (mo)	Clinical feature	Eye	Laboratory tests (U)	Type	Intervention	Initial visual acuity	Visual outcome	Duration of follow-up (mo)
1	15	F	2	Posterior granuloma, ERM, TRD	Left	Aqueous humor: 74.91 Serum: 44.89	Posterior pole granuloma	IV, PPV, membrane removal	20/200	20/100	31

2	22	M	36	Peripheral granuloma, TRD, peripheral traction band	Left	Aqueous humor: 43.86 Serum: 35.60	Peripheral granuloma	IV, PPV, membrane removal, SB	LP	20/100	17

3	12	M	5	Vitritis	Left	Aqueous humor: 66.78 Serum: 66.81	Endophthalmitis	IV, cataract operation	20/200	20/20	18

4	11	F	12	Peripheral granuloma, ERM, peripheral traction band	Right	Aqueous humor: 57.84 Serum: 41.60	Peripheral granuloma	IV, SB	CF	20/40	12

5	29	F	6	Vitritis	Right	Aqueous humor: 69.90 Serum: 45.66	Endophthalmitis	IV, PPV, membrane removal, oil	20/100	20/40	16

6	5	F	2	Posterior granuloma, ERM, TRD	Right	Aqueous humor: 31.07 Serum: 19.97	Posterior pole granuloma	IV, PPV, membrane removal, oil	HM	20/400	24

RD, retinal detachment; TRD, traction retinal detachment; ERM, epiretinal membrane; IV, intravitreal injection; PPV, pars plana vitrectomy; SB, scleral buckle; HM, hand motions; LP, light perception; CF, count fingers.

## Data Availability

The data that support the findings of this study are available from the corresponding author upon reasonable request.
